# A mathematical model for Zika virus transmission dynamics with a time-dependent mosquito biting rate

**DOI:** 10.1186/s12976-018-0083-z

**Published:** 2018-08-01

**Authors:** Parinya Suparit, Anuwat Wiratsudakul, Charin Modchang

**Affiliations:** 1 Biophysics Group, Department of Physics, Faculty of Science, Mahidol University, Bangkok, 10400 Thailand; 2 Department of Clinical Sciences and Public Health, Faculty of Veterinary Science, Mahidol University, Phutthamonthon, Nakhon Pathom, 73170 Thailand; 3 The Monitoring and Surveillance Center for Zoonotic Diseases in Wildlife and Exotic Animals, Faculty of Veterinary Science, Mahidol University, Phutthamonthon, Nakhon Pathom, 73170 Thailand; 4 Centre of Excellence in Mathematics, CHE, Bangkok, 10400 Thailand; 5 Thailand Center of Excellence in Physics, CHE, 328 Si Ayutthaya Road, Bangkok, 10400 Thailand

**Keywords:** Zika virus, Climatic factors, Brazil, Vector control, Mosquito biting rate

## Abstract

**Background:**

Mathematical modeling has become a tool used to address many emerging diseases. One of the most basic and popular modeling frameworks is the compartmental model. Unfortunately, most of the available compartmental models developed for Zika virus (ZIKV) transmission were designed to describe and reconstruct only past, short-time ZIKV outbreaks in which the effects of seasonal change to entomological parameters can be ignored. To make an accurate long-term prediction of ZIKV transmission, the inclusion of seasonal effects into an epidemic model is unavoidable.

**Methods:**

We developed a vector-borne compartmental model to analyze the spread of the ZIKV during the 2015–2016 outbreaks in Bahia, Brazil and to investigate the impact of two vector control strategies, namely, reducing mosquito biting rates and reducing mosquito population size. The model considered the influences of seasonal change on the ZIKV transmission dynamics via the time-varying mosquito biting rate. The model was also validated by comparing the model prediction with reported data that were not used to calibrate the model.

**Results:**

We found that the model can give a very good fit between the simulation results and the reported Zika cases in Bahia (R-square = 0.9989). At the end of 2016, the total number of ZIKV infected people was predicted to be 1.2087 million. The model also predicted that there would not be a large outbreak from May 2016 to December 2016 due to the decrease of the susceptible pool. Implementing disease mitigation by reducing the mosquito biting rates was found to be more effective than reducing the mosquito population size. Finally, the correlation between the time series of estimated mosquito biting rates and the average temperature was also suggested.

**Conclusions:**

The proposed ZIKV transmission model together with the estimated weekly biting rates can reconstruct the past long-time multi-peak ZIKV outbreaks in Bahia.

**Electronic supplementary material:**

The online version of this article (10.1186/s12976-018-0083-z) contains supplementary material, which is available to authorized users.

## Background

The Zika virus (ZIKV) was first recovered from a Rhesus monkey during a research study on Yellow fever in Zika Forest, Uganda in 1947 [1]. The virus was subsequently isolated from *Aedes* mosquitoes and humans in 1948 and 1954, respectively [1, 2]. ZIKV is a Flavivirus belonging to the family of *Flaviviridae* [3]. The virus is closely related to many other well-known notorious pathogen causing encephalitis viruses such as Dengue, Japanese encephalitis and West Nile virus [4]. Two species of mosquitoes, namely, *Aedes aegypti* and *Aedes albopictus,* were identified as the main vectors for ZIKV transmission [5].

Epidemiologically, ZIKV cases were only sporadically recorded in some African and Southeast Asian countries until the late 2000s [6]. In 2013, the first large scale outbreaks of ZIKV was observed in French Polynesia with the evidence of ZIKV related Guillain–Barré syndrome. Over 19,000 suspected cases were estimated during this epidemic [7]. Since then, the expansion of the outbreaks seems unstoppable. The situation was worse when the virus reached and became well-established in Latin America; Brazil is one of the most affected countries. The number of suspected cases in Brazil was estimated at 440,000 to 1,300,000 in 2015 [8]. Anxiously, the increase of microcephaly incidence was unexpectedly observed in the outbreaks [9]. Hence, the World Health Organization (WHO) decided to elevate the ZIKV epidemic status to the level of “a Public Health Emergency of International Concern (PHEIC)” on February 1, 2016 [10].

*Aedes aegypti* and *Aedes albopictus* seem to have different biological lifestyles, feeding preferences, and susceptibilities to ZIKV [11, 12]. *Aedes aegypti* extensively feeds on human blood [13] whereas *Aedes albopictus* feeds on a more variety of host species [14]. Both species are diurnal feeders providing high chance to expose and bite humans. *Aedes aegypti* basically breeds in manmade containers such as jars and old tires while *Aedes albopictus* may also extend the breeding sites to some other natural water holders, for examples, tree holes and coconut shells [15]. The eggs of *Aedes aegypti* can survive the dry period over 8 months [14]. However, cold egg diapause is poorly known for this species. In contrast, the evidences were obvious that *Aedes albopictus* found in temperate environment can produce diapausing eggs and survive hard winter resulting in a wider geographical distribution [16].

Weather and climate conditions are known to affect the transmission dynamics of vector-borne diseases through the modulation of entomological parameters, e.g., mosquito population abundance, lifespan, biting rates, and extrinsic incubation period (the time required for development of virus inside a mosquito) [17–22]. In particular, temperature is believed to be a major driver of ZIKV transmission [17, 19]. Both field and laboratory experiments demonstrate that survival of both *Aedes aegypti* and *Aedes albopictus* is affected by temperature [23]. Data gathered from the literature also reveal that biting rate respond strongly to change in temperature [17]. In addition, a computational analysis also showed that the basic reproduction number of vector-borne diseases is very sensitive to change in the mosquito biting rate [24]. Therefore, to make an accurate long-term prediction of ZIKV transmission, the inclusion of seasonal effects into an epidemic model is unavoidable.

Mathematical modeling has become a tool used to address many emerging diseases. One of the most basic and most popular modeling frameworks is the compartmental model. The model divides the population of interest based on their health status [25], for example, susceptible (S), exposed (E), infectious (I) and recovered (R). The compartmental model has been employed in the study of transmission dynamics in many vector-borne diseases such as Dengue [26, 27] and Malaria [28]. In the ZIKV study, this modeling architecture has also been constructed [29–33]. Unfortunately, most of the available mathematical models developed for ZIKV transmission were designed to describe and reconstruct only past, short-time ZIKV outbreaks in which the effects of seasonal change to entomological parameters can be ignored [30–33]. Although a more sophisticated stochastic individual-based model incorporating high-resolution demographic, human mobility, and temperature data exists [34], it requires time to develop and implement this kind of complicated model. For a more comprehensive review of mathematical models developed for ZIKV transmission, we refer the reader to the recent work by Wiratsudakul et al. (2018) [35].

The present work, therefore, aimed to construct a simple vector-borne compartmental model that can reconstruct the past long-time ZIKV transmission and project the future spread of ZIKV. Since it was found that the transmission of a vector-borne disease is most sensitive to change in the mosquito biting rate [24], we therefore integrated effects of seasonal change with mosquito biting rates in the model. Values of unknown entomological parameters were also estimated with a computational parameter estimation algorithm. The effectiveness of two vector control strategies, namely, reducing mosquito biting rates (e.g., via repellents) and reducing mosquito population size (e.g., via adulticides, larvicides), were investigated. Finally, a possible correlation between the estimated mosquito biting rates and temperature was also discussed.

## Methods

### Mathematical model

**Fig. 1:**
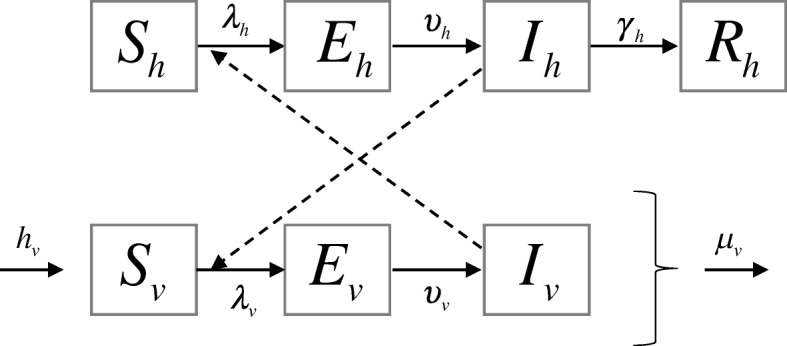
Schematic of the ZIKV transmission model. The solid arrows represent transitions between epidemiological classes, whereas the dash arrows represent interactions between humans and mosquitoes. Susceptible humans (*S*_*h*_) can be infected if they are bitten by infectious mosquitoes. Infected humans immediately become exposed (*E*_*h*_) and then transition to the infectious class (*I*_*h*_) after the intrinsic incubation period. Finally, infectious humans move to recovered class (*R*_*h*_). Mosquitoes enter the susceptible class at a birth rate *h*_*v*_ and die at a natural death rate *μ*_*v*_. If susceptible mosquitoes (*S*_*v*_) bite infectious humans, they are moved to the exposed (*E*_*v*_) class and then become infectious (*I*_*v*_)

Figure 1 describes the compartmental classifications used to simulate ZIKV transmission dynamics. The model divides the population into two subpopulations, namely, the human and mosquito vector populations. A modeling study shows that either considering both *Aedes aegypti* and *Aedes albopictus* as competent ZIKV vectors or considering *Aedes aegypti* as the only competent vector give similar modelling results [34]. To simplify the model, we therefore consider *Aedes aegypti* as the only competent ZIKV vector. Within each subpopulation, a compartmental model is employed to simulate ZIKV transmission while an interaction between subpopulations occurs through the mosquito bites. Human population is classified into the following 4 epidemiological classes: susceptible (*S*_*h*_), exposed (*E*_*h*_), infectious (*I*_*h*_), and recovered (*R*_*h*_), whereas mosquito vector population is divided into the following 3 epidemiological classes: susceptible (*S*_*v*_), exposed (*E*_*v*_), and infectious (*I*_*v*_). When an infectious mosquito bites a susceptible human, ZIKV can be transmitted to the human under the mosquito-to-human force of infection *λ*_*h*_. After being infected, the susceptible human progresses to the exposed class. Humans in this class have already acquired the infection, but are not yet infectious and cannot transmit ZIKV to a mosquito. Exposed humans become infectious at a rate *υ*_*h*_, which is inversely proportional to the intrinsic incubation period. During this stage of infection, they can transmit ZIKV to a susceptible mosquito through biting. These infectious individuals then recover from the disease and have lifelong immunity at a rate that is inversely proportional to the infectious period, *γ*_*h*_. In this model, we assumed that the dynamics of human birth and death are much slower than the dynamics of the epidemic. Therefore, they are omitted. Thus, the human population size (*N*_*h*_) is constant, that is, *S*_*h*_ + *E*_*h*_ + *I*_*h*_ + *R*_*h*_ = *N*_*h*_.

Similarly, susceptible mosquitoes can be infected under the human-to-mosquito force of infection *λ*_*v*_ if they bite an infectious human. After being infected, the susceptible mosquitoes transition to the exposed class. These exposed mosquitoes can then become infectious at a rate *υ*_*v*_, which is inversely proportional to the extrinsic incubation period. Unlike humans, the infectious mosquitoes are assumed to remain infectious for life. Mosquitoes in all epidemiological classes die at a natural death rate, *μ*_*v*_, that is inversely proportional to the mosquito lifespan. In this model, we assume that ZIKV does not affect the mosquito lifespan [24]. All newborn mosquitoes are assumed to be susceptible and enter the susceptible class at a birth rate *h*_*v*_.

Based on the law of mass action, the dynamics of ZIKV transmission are described by the following ordinary differential equations:

$$ \frac{dS_h}{dt}=-{\lambda}_h(t){S}_h $$

$$ \frac{dE_h}{dt}={\lambda}_h(t){S}_h-{\upsilon}_h{E}_h $$

$$ \frac{dI_h}{dt}={\upsilon}_h{E}_h-{\gamma}_h{I}_h $$

$$ \frac{dR_h}{dt}={\gamma}_h{I}_h $$

$$ \frac{dS_v}{dt}={h}_v{N}_v-{\lambda}_v(t){S}_v-{\mu}_v{S}_v $$

$$ \frac{dE_v}{dt}={\lambda}_v(t){S}_v-{\upsilon}_v{E}_v-{\mu}_v{E}_v $$

$$ \frac{dI_v}{dt}={\upsilon}_v{E}_v-{\mu}_v{I}_v $$

where *N*_*v*_ = *S*_*v*_ + *E*_*v*_ + *I*_*v*_ is the mosquito population size. Following [24, 36], the forces of infection are assumed to be

$$ {\lambda}_h=\frac{\sigma_v{\sigma}_h{N}_v}{\sigma_v{N}_v+{\sigma}_h{N}_h}{\beta}_{hv}\frac{I_v}{N_v} $$

$$ {\lambda}_v=\frac{\sigma_v{\sigma}_h{N}_h}{\sigma_v{N}_v+{\sigma}_h{N}_h}{\beta}_{vh}\frac{I_h}{N_h} $$

where *σ*_*v*_ represents the number of times a mosquito can bite humans per unit time, and *σ*_*h*_ describes the maximum number of mosquito bites that a human can support per unit time. If an infectious mosquito bites a susceptible human, ZIKV can be successfully transmitted to a human with a probability *β*_*hv*_, whereas if a susceptible mosquito bites an infectious human, ZIKV can be transmitted to a mosquito with a probability *β*_*vh*_. The descriptions and values of all parameters used in the model are summarized in Table 1.

**Table 1:** Descriptions and values of all parameters used in the model

Parameter	Definition	Value	Reference
*σ*_*h*_	Maximum number of bites a human can sustain	19 bites/day	[24]
*σ*_*v*_	Mosquito biting rate	See estimation in result section	
*β*_*hv*_	Probability of pathogen transmission from an infectious mosquito to a susceptible human	0.7	[29]
*β*_*vh*_	Probability of pathogen transmission from an infectious human to a susceptible mosquito	0.7	[29]
*υ*_*h*_	Human progression rate from exposed state to infectious state	1/5.5 per day	[29]
*υ*_*v*_	Mosquito progression rate from exposed state to infectious state	1/8.2 per day	[29]
*γ*_*h*_	Human recovery rate	1/6 per day	[29]
*h*_*v*_	Mosquito birth rate	1/14 per day	[24]
*μ*_*v*_	Mosquito natural death rate	1/14 per day	[24]
*N*_*h*_	Human population size in Bahia, Brazil	15,276,566	[29]
*N*_*v*_	Mosquito population size	10–250 million	Assumed

### Estimated time series of weekly mosquito biting rates

We estimated the time series of weekly biting rates that best fit the modeling results to the reported data. The estimation was performed by dividing the model simulation into several consecutive weekly time-window simulations. The model searched for a weekly biting rate that gives the best fit between the simulation result and the corresponding weekly reported data. Mathematically, we searched for a value of weekly biting rate (*σ*_*v*_) that minimizes |*C*_*sim*_ − *C*_*rep*_|, which is the absolute difference between number of weekly reported cases (*C*_*rep*_) and number of weekly cases simulated from the model (*C*_*sim*_). The number of weekly simulated cases was calculated using the equation ${C}_{sim}=\frac{dI_h}{dt}+\frac{dR_h}{dt}$. Possible values of weekly biting rates that the model can search for is uniformly distributed in the interval [0, 1] with 0.01 resolution. Only the value of biting rate that yields the best simulation result was recorded. The simulation result, i.e., the number of humans and mosquitoes in each epidemiological class, for the current weekly time-window was then used as an initial condition for the next weekly time-window simulation. The search process was repeated until the complete time series of the weekly biting rates was obtained.

### Data

The reported weekly Zika cases were derived from the data presented in the literature [29, 37]. To minimize consequences that may have resulted from the difference in spatial temperature profiles, the ZIKV transmission dynamics were simulated in the smallest possible spatial scale, i.e., city or state. Since the first reported Zika case in Brazil was in the state of Bahia [38] and the outbreaks in Bahia show the most prominent state-level outbreaks (about 64% of ZIKV infections in Brazil occurred in Bahia) [29], we therefore used the Bahia outbreak data to calibrate the model. The total number of reported cases, which is the sum of the laboratory-confirmed cases and the suspected cases, was used in the present work. The reported weekly Zika cases in Bahia from 1st January 2015 to 18th May 2016 were used in estimating the time series of weekly biting rates [29]. Since there was no officially released data of Zika cases in the state of Bahia after May 2016, we therefore used official information at the national level to compare with the model prediction during June 2016 – December 2016. The numbers of weekly Zika cases in Brazil during June 2016 – December 2016 were derived from reference [37]. Data presented in [37] was only in graphical forms, the WebDigitizer tool (https://automeris.io/WebPlotDigitizer/) was used to extract the approximate number of reported cases. Because ZIKV transmission dynamics in Bahia and Brazil are different in terms of population size and spatiotemporal scale, e.g., the peak value of weekly Zika cases in Brazil is 21,235 while the peak value of weekly Zika cases in Bahia is 6823 [29, 37], for a more appropriate comparison with the model prediction, we therefore rescaled the number of weekly Zika cases in Brazil by the ratio of peak values of Zika cases in Bahia and Brazil, i.e., 6823/21,235 = 0.32. This rescaling makes the two data sets to have the same maximum value. The monthly average temperature in Bahia was derived from reference [39].

## Results

### Time series of weekly mosquito biting rates

**Fig. 2:**
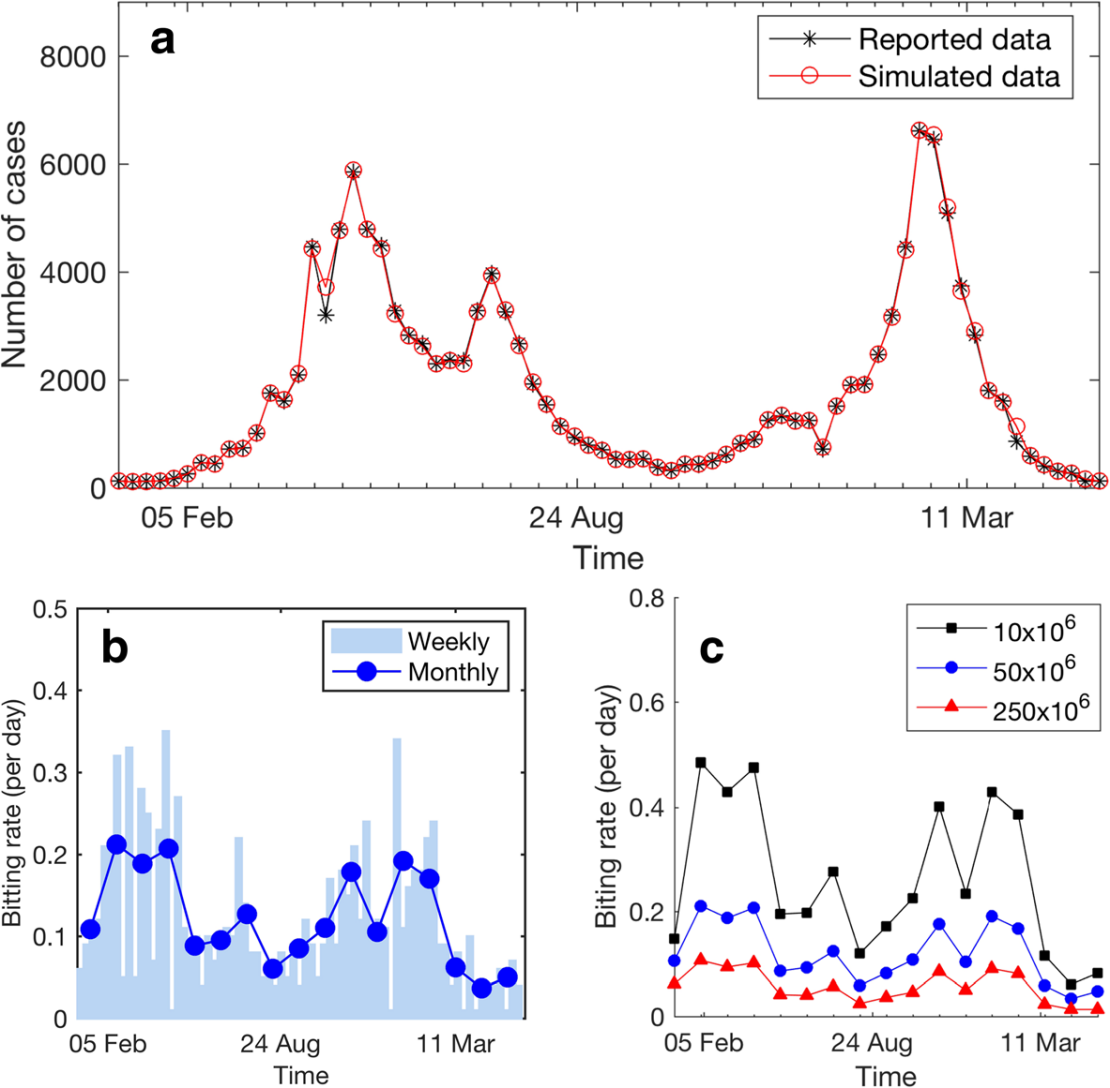
Model fitting and mosquito biting rates. **a** The fit of the simulation results and the reported data with R-square = 0.9989. The weekly reported data of Zika cases in Bahia were adapted from the data presented in reference [29]. The simulation results obtained by searching for the best weekly biting rates are shown as a red solid line (○). **b** The estimated weekly biting rates (bars) overlaid with monthly average biting rates (line). **c** Plots of the monthly average biting rates obtained using the mosquito population size of 10, 50, and 250 million

The reported weekly Zika cases in Bahia were used to estimate the time series of weekly biting rates. The reported data begin in the 1st week of 2015 and end in the 22nd week of 2016. The reported data shows two episodes of epidemics in Bahia (Fig. 2a). The first epidemic peak appears around May 2015, and the second peak occurs around March 2016. As seen in Fig. 2a, the proposed biting rate estimation algorithm provides a very good fit of the simulation results and the reported data (R-square = 0.9989). Based on the estimation algorithm, the weekly biting rates were found to be in the range of 0.01–0.35 bites per day (Fig. 2b, bars). In order to reduce variation in estimated weekly biting rates that may have resulted from random variation in weekly reported data, monthly average biting rates are also presented (Fig. 2b and c), solid lines). Unless stated otherwise, the simulations reported assume that the mosquito population size is 50 million. However, a sensitivity analysis of the mosquito population size was also performed. By changing the assumed initial number of mosquitoes, we found that a 25-fold change in the number of mosquitoes results in approximately a 4-fold change in the estimated biting rates (Fig. 2c). This indicates that the ZIKV transmission dynamics are less sensitive to the change in the mosquito population size than the change in mosquito biting rate.

### Projected ZIKV infections and model validation

**Fig. 3:**
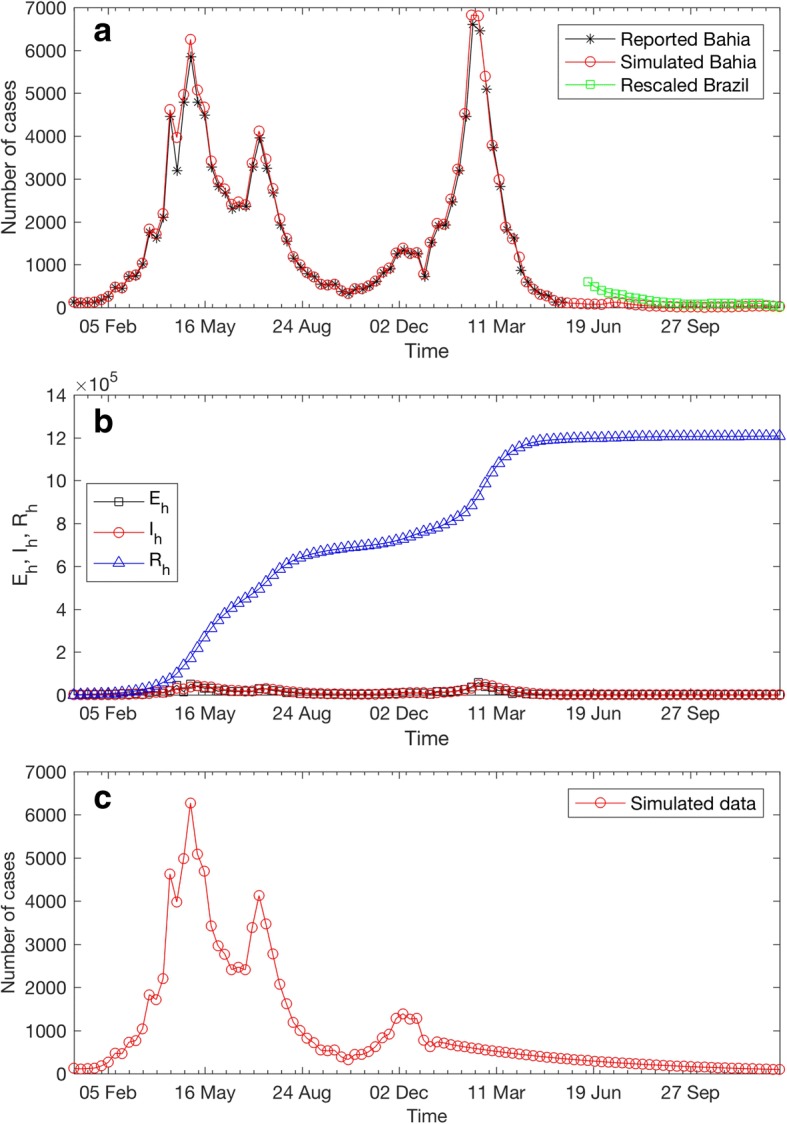
Model projections. **a** The predicted number of weekly ZIKV infection cases from 1 January 2015 to 31 December 2016 in Bahia (○), and the rescaled weekly reported ZIKV cases from June 2016 – December 2016 in Brazil (□) [37]. **b** Predicted number of humans in the exposed (□), infectious (○) and recovered (△) classes. Note that the number of humans in each epidemiological class was obtained by multiplying with the under report factor of 11.5% [31]. **c** The predicted number of weekly ZIKV infection cases when the seasonal variation of biting rates in year 2016 was removed

The projection of ZIKV infections in Bahia was done by assuming that the time series of weekly biting rates are yearly periodic [40, 41]. In this section, we therefore use the time series of weekly biting rates from May 2015 to December 2015 to project the ZIKV transmission during May 2016 to December 2016. We also validated our model by comparing the model projections with reported data that were not used for its calibration. The projection results are shown in Fig. 3. It can be clearly seen in Fig. 3a that the model predicts no large outbreak after May 2016, a finding supported by epidemiological surveillance in Brazil [37]. Specifically, the model predicts that there will be 43 average weekly cases during June–December 2016. This number is close to 157 average weekly cases calculated using the rescaled Brazil data (green line in Fig. 3a) [37]. The predicted numbers of humans in exposed, infectious, and recovered classes are shown in Fig. 3b. Note that the numbers of humans shown in Fig. 3b were multiplied with the under report factor of 11.5% [31]. At the end of 2016, the cumulative number of infected people was predicted to be 1.2087 million. We then further investigated if the observed decrease in weekly cases after May 2016 occurred due to the depletion of the susceptible pool. We performed a sensitivity analysis in which the seasonal variation of biting rates in year 2016 was removed. This was accomplished by replacing the time series of estimated biting rates in year 2016 with its average value (0.12 bite/day). As shown in Fig. 3c, it can be clearly seen that despite the seasonal variation in biting rates had been removed, the model still predicts a decrease in weekly cases after the large outbreaks in year 2015. We therefore suspect that the decrease in number of infections in Bahia after May 2016 may be due to the depletion of susceptible individuals.

### Effects of reducing mosquito biting rate

**Fig. 4:**
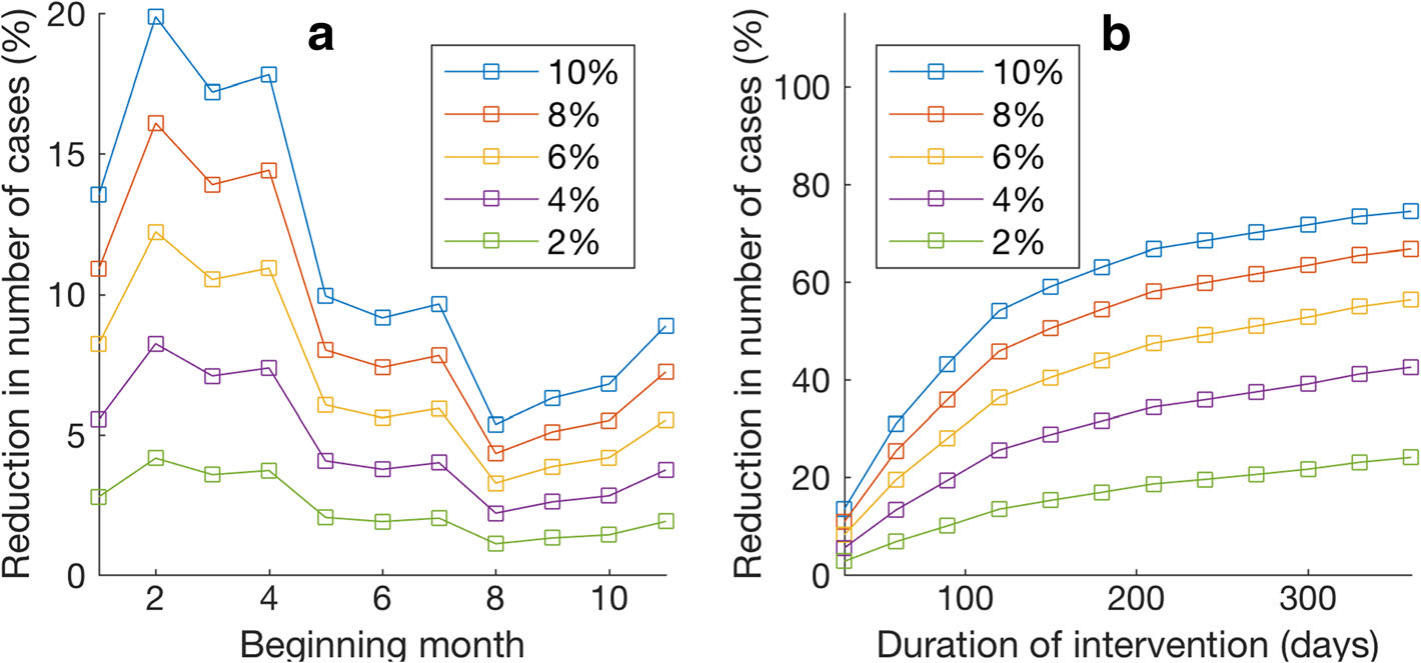
Effects of reducing the mosquito biting rates. **a** The percentage of reduction in the total number of ZIKV cases when the intervention was implemented for 30 days starting at different times. The *x*-axis indicates a month (from 1st - 11th month of 2015), in which the vector control began. Each curve shows the result when different percentage of biting rate reduction was implemented. **b** The percentage of reduction in total number of ZIKV cases when the intervention was started in January 2015 and was implemented for a different duration. The *x*-axis indicates the length of time in which the intervention was implemented, and each curve shows the result when different percentages of biting rate reduction were implemented

We further used the developed ZIKV transmission model and the estimated parameters to investigate the effectiveness of two vector control strategies, namely, reducing mosquito biting rates and reducing mosquito population size. To investigate the effects of temporarily reducing mosquito biting rates, the ZIKV transmission model was simulated with reduced mosquito biting rates during a specified period while keeping other parameters unchanged. Figure 4a shows the percentage of reduction in number of cumulative ZIKV cases when the mosquito biting rates were reduced for 30 days during a different period. We found that the biting rate reduction strategy yields the best result when implemented during the 2nd - 4th months of 2015, which is approximately the period in which the epidemic reaches its first peak. Implementing the biting rate reduction strategy after this period will lower the intervention effectiveness. From the results, we can see that, for example, reducing the mosquito biting rates by 10% for 30 days can reduce the cumulative number of ZIKV cases up to 20%. In addition, Fig. 4b shows effects of implementing the biting rate reduction strategy for a different length of time beginning with the first month of 2015; reducing the mosquito biting rate by 10% for 3 months is enough to reduce the number of total infections by half.

### Effects of reducing mosquito population size

**Fig. 5:**
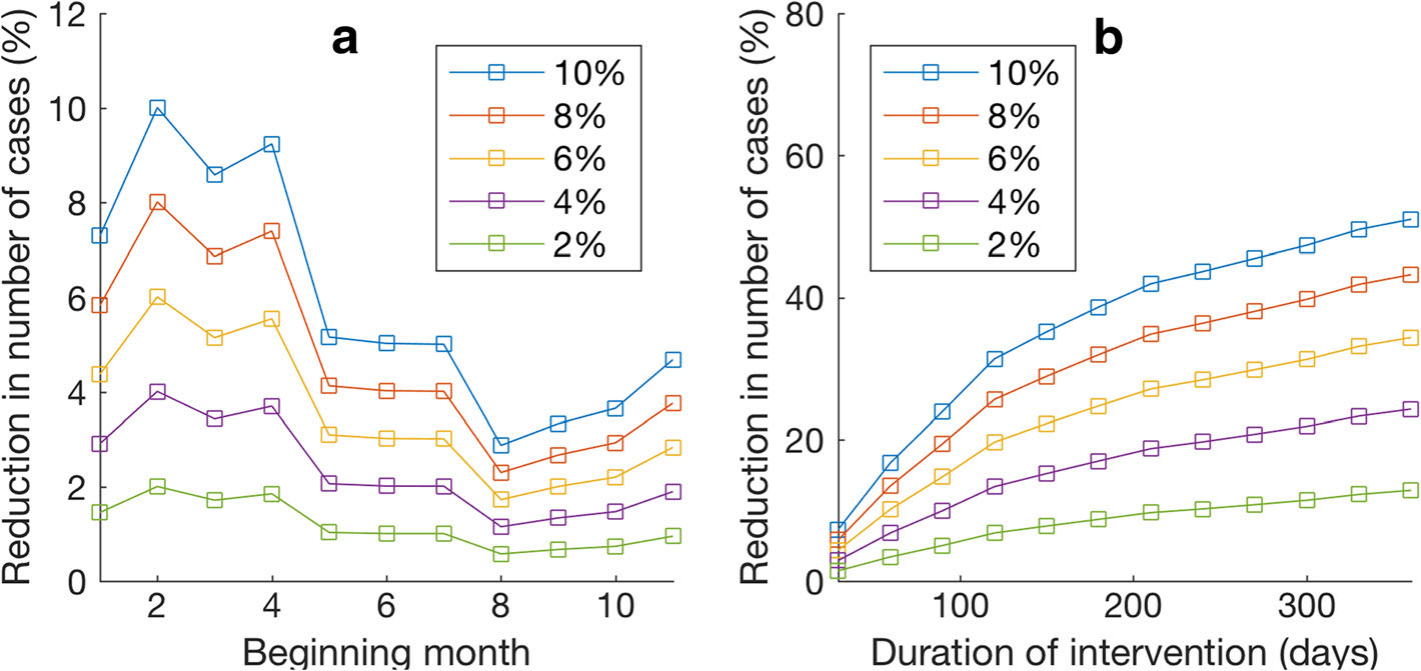
Effects of reducing the mosquito population size. **a** The percentage of reduction in total number of ZIKV infections when the mosquito control strategy was implemented for a duration of 30 days and started at a different time. The *x*-axis indicates a month (from the 1st - 11th month of 2015) in which the intervention was started. Each curve shows the result when different percentages of population size reduction were implemented. **b** The percentage of reduction in total number of ZIKV cases when the intervention was started at January 2015 and was implemented for a different length of time. The *x*-axis indicates the length of time in which the intervention was implemented, and each curve shows the result when different percentages of mosquito population size reduction were implemented

Since reducing the number of mosquitoes can also decrease the number of contacts between humans and mosquitoes, this may also reduce the epidemic impact. Hence, to investigate the effects of temporarily reducing mosquito population size, we employed similar methodology as in the previous section. The ZIKV transmission model was simulated with a reduced mosquito population size during a specified period while other parameters remained unchanged. Figure 5a shows the percentage of reduction in total ZIKV infections when the mosquito population size was reduced for a duration of 30 days with at a different start time; reducing the mosquito population size by 10% can reduce the number of infections by a maximum of 10%. Implementing the intervention strategy at a different time results in different intervention effectiveness. We found that the intervention yields the best result when it was completed during the 2nd - 4th months of 2015. Obviously, implementing the vector control strategy for a longer time can further decrease the total number of infections. Figure 5b shows the percentage of reduction in the number of ZIKV cases when the mosquito population size was reduced for a different length of time, starting in the first month of 2015.

### Correlation between mosquito biting rates and temperature

**Fig. 6:**
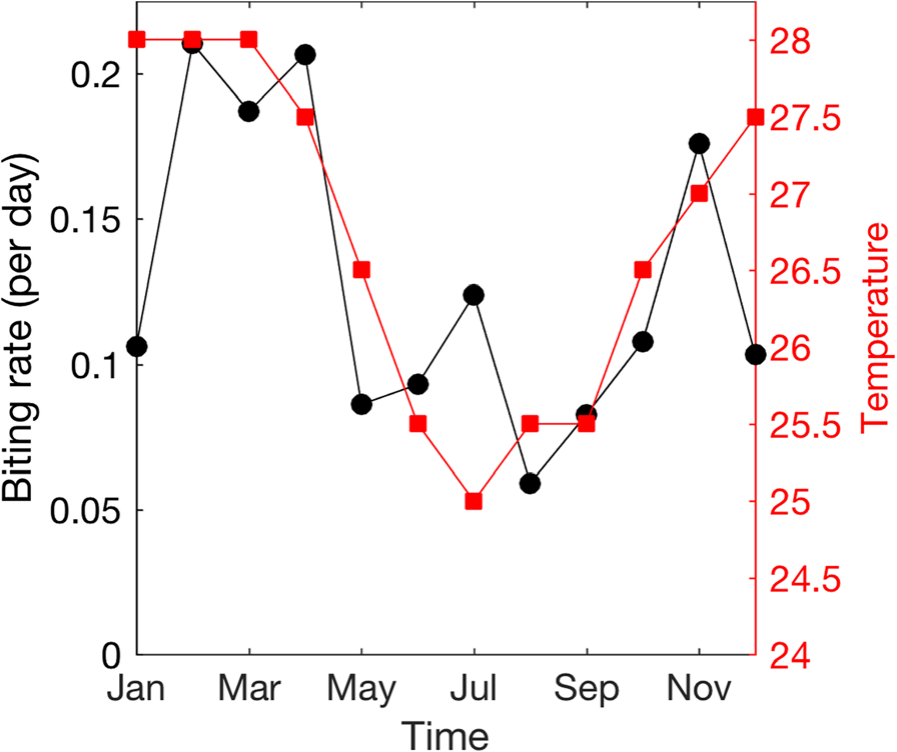
Correlation between monthly average biting rate and monthly average temperature. The monthly average biting rates and monthly average temperature in Bahia during January 2015 – December 2015 are plotted on the same graph. The left y-axis shows the monthly average biting rates (shown as black circles), and the right y-axis indicates the monthly average temperature in degree Celsius (shown as red squares) [39]

To see the correlation between the estimated mosquito biting rates and the average temperature, the monthly average biting rates and the monthly average temperature in Bahia during January 2015 – December 2015 are plotted and compared on the same graph (Fig. 6). The trend of monthly average biting rates qualitatively agrees with the trend of monthly average temperature; the values reach maximum and minimum points at roughly the same times. Quantitatively, the Pearson correlation coefficient between the monthly average biting rates and monthly average temperature was found to be 0.6404 with the *p*-value of 0.0249 and the Spearman’s rank correlation coefficient was 0.5980 with the p-value of 0.0400. In these results, both Pearson correlation coefficient and Spearman’s rank correlation coefficient indicate that there is a moderate positive relationship between the monthly average biting rates and monthly average temperature. In addition, the *p*-values for both correlation coefficients are less than the significance level of 0.05, which indicates that the correlation coefficients are significant. Both correlation coefficients were calculated using the command *corr* in MATLAB R2017a.

## Discussion

We proposed a mathematical model and used computer simulations to describe ZIKV transmission in Bahia. The aims of the simulations were to reconstruct the past long-time multi-peak ZIKV outbreaks and project the future spread of ZIKV, as well as to investigate the impact of the vector control strategies. Climate factors are known to affect the transmission dynamics of vector-borne diseases [17–22], to accurately reconstruct long-time multi-peak ZIKV outbreaks and to make an accurate long-term prediction of ZIKV transmission, the inclusion of seasonal effects into the epidemic model is unavoidable. Although climate conditions can affect to several entomological parameters including larval carrying capacity, extrinsic incubation period, mosquito lifespan, and biting rate [17–22], however, a mathematical analysis shows that a vector-borne disease transmission is most sensitive to change in the mosquito biting rate [24]. We therefore hypothesized that seasonal changes in mosquito biting rates mainly contributed to the two ZIKV epidemic peaks in Bahia during the years 2015–2016. To reconstruct the ZIKV transmission in Bahia, we therefore estimated the time series of mosquito weekly biting rates that best fit the modeling results to the reported data from 1st January 2015 to 18th May 2016. The time variations of the estimated mosquito biting rates were also compared with the seasonal changes of the average temperature in Bahia. In contrast to previous ZIKV transmission models [29–33], we believe that our Zika epidemic model is the first compartmental model that can accurately simulate two peaks of ZIKV outbreaks in Bahia (please see reference [35] for a comprehensive review of ZIKV transmission models).

The model fitting results (Fig. 2a) show that the proposed ZIKV transmission model together with the estimated time series of weekly biting rates give a very good fit between the simulation results and the reported data (R-square = 0.9989). Although seasonal environmental factors can affect to several entomological parameters [17–22], our modeling results suggested that only assuming the mosquito biting rates to be dependent on seasonal climate may be sufficient for modeling proposes. This may be because the transmission of vector borne diseases is most sensitive to the mosquito biting rate [24]. Note, however, that our estimated mosquito biting rates may already implicitly compensate for the effects of seasonal changes on other entomological parameters. In addition, since we considered *Aedes aegypti* as the only competent ZIKV vector in our model, the estimated biting rates may also compensate for the absence of the *Aedes albopictus* vector [34]. Since the actual mosquito population size in Bahia is not available, we also varied the mosquito population size in the simulations. We found that the dynamics of ZIKV transmission is less sensitive to the change in the mosquito population size than the change in the mosquito biting rate. The 25-fold change in the number of mosquitoes is equivalent to an approximate 4-fold change in biting rates (Fig. 2c).

We also validated our model by comparing the model projections with reported data that were not used to calibrate the model. By assuming that the time series of weekly biting rates are yearly periodic, we then used the ZIKV transmission model and the estimated parameters to project the ZIKV infections from May 2016 to December 2016 (Fig. 3a-b). We found that the model predicts no large outbreaks during May 2016 to December 2016. This finding agrees with the data from epidemiological surveillance in Brazil [37]. Specifically, the model predicts that there will be 43 average weekly cases during June–December 2016. This number is close to 157 average weekly cases as calculated using the rescaled Brazil data [37]. The decrease in number of infections in the model may be largely due to the fact that large outbreaks in 2015–2016 greatly deplete the pool of susceptible individuals who can be exposed to the disease. The model suggests that herd immunity may have already been achieved, a finding supported by a more complex modelling study [29]. The herd immunity may cause a delay of more than a decade until further large outbreaks are possible [29]. However, like other mosquito-borne viral diseases, we may expect small seasonal oscillations in ZIKV incidence caused by seasonal temperature variation [29, 42, 43]. Nevertheless, it is possible that the decrease in the number of notified ZIKV cases in 2016 was caused by a competition of ZIKV and other arboviruses that share the same invertebrate and vertebrate hosts [44]. A recent study indicated that the introduction of chikungunya virus (CHIKV) in the Recife Metropolitan Region in northeast Brazil helped to suppress the circulation of ZIKV in the area [44]. There is an estimate that only 11.5% of the total ZIKV infections in the 2013–2014 French Polynesia outbreak were reported [31]. Considering this, the numbers of human individuals in each epidemiological class predicted from the model were multiplied with 100/11.5 (Fig. 3b). We found that at the end of 2016, the total number of infected people was 1.2087 million, which is consistent with the number from the other estimate [8]. Note, however, that the actual underreport factor in the Bahia outbreak may be different from that of the French Polynesia outbreak.

In this work, two vector control strategies, namely, reducing mosquito biting rates (e.g., via repellents) and reducing mosquito population size (e.g., via adulticides, larvicides, or other methods) were investigated. We found that the mitigation strategy that affects the more sensitive parameters have a larger impact on the magnitude of the epidemic. Specifically, we found that reducing the mosquito biting rates by 10% for 30 days can reduce ZIKV infections up to 20% (Fig. 4); however, reducing the mosquito population size by 10% for 30 days can reduce the number of infections by a maximum of 10% (Fig. 5). Implementing the vector control strategy at a different time also results in different intervention effectiveness. We found that either reducing the mosquito biting rate or reducing the mosquito population size yields the best result when implemented during the first peak of the epidemic.

Several studies have examined the impacts of temperature on the transmission of vector borne diseases [17, 21, 22]. In this work, the correlation between the estimated time series of mosquito biting rates and average temperature was investigated. We found that the trend of monthly average biting rates qualitatively agrees with the trend of monthly average temperature (Fig. 6). Although some studies [29, 34] assumed that seasonal changes may affect several entomological parameters, e.g., larval carrying capacity, extrinsic incubation period, mosquito lifespan, and biting rate, our modeling results suggested that only assuming the mosquito biting rates to be dependent on temperature may be sufficient for modeling proposes. This may be because the basic reproduction number of vector borne diseases is most sensitive to the mosquito biting rate [24].

The ZIKV transmission model presented here also has some limitations. The model assumes that ZIKV behaves similarly to other mosquito borne disease viruses, e.g., dengue virus and chikungunya virus. We assumed all human and mosquito individuals are homogeneously mixed and that all individuals have equal chance of contact; however, in reality, there may be spatial heterogeneity in contact and disease transmission [45]. The sexual transmission route of ZIKV is not considered in the proposed model, but a mathematical modeling analysis indicated that transmission contributed by sexual activity is small [30].

Although vector control strategies, e.g., reducing mosquito population size and mosquito biting rate, can reduce ZIKV transmission, adherence to these intervention strategies over the long term in practice can be very difficult to achieved. A ZIKV vaccine may be the best way to protect at-risk populations over the long term [46, 47]. It is worthwhile to mention that our ZIKV transmission model may be extended to incorporate vaccinated compartments and employed to assess the effectiveness of vaccination strategies. This should be a potential topic for future research.

## Conclusions

We constructed a mathematical model for fitting the reported Zika cases in Bahia, Brazil, during the 2015–2016 outbreaks, predicting the possible future spread of ZIKV and investigating the impact of vector control strategies. The ZIKV transmission model integrates the effects of seasonal change into mosquito biting rates, which were estimated using a computational parameter estimation algorithm. We found that the ZIKV transmission model together with the estimated weekly biting rates gives a very good fit between the simulation results and the reported data (R-square = 0.9989). The model predicted the total number of infected people at the end of 2016 to be 1.2087 million, which is close to the estimated numbers from a previous study [8]. We also found that implementing the vector control strategy that affects the most sensitive parameter can have a large impact on the magnitude of the epidemic, i.e., reducing the mosquito biting rates by 10% for 30 days can reduce ZIKV infections up to 20% whereas reducing the mosquito population size by 10% for 30 days can reduce the number of infections by 10% at most. The estimated mosquito biting rates were also found to be correlated with the average temperature with the Pearson correlation coefficient of 0.6404 (*p*-value: 0.0249) and the Spearman’s rank correlation coefficient of 0.5980 (*p*-value: 0.0400).

## Additional file

Additional file 1: The main code for estimating the weekly biting rates and running the simulation is *Estimate_Biting_Rate_Main_Code.m* and *Main_Code.m*, respectively. (7Z 3143 kb)

## Abbreviation

ZIKV: Zika virus
